# Self-reported hopefulness and cognitive function: the moderating effect of physical activity in older adults without cognitive impairment

**DOI:** 10.3389/fnagi.2025.1646298

**Published:** 2025-11-14

**Authors:** Boung Chul Lee, Young Min Choe, Ji-Hyun Kim, Hye Ji Choi, Guk-Hee Suh, Shin Gyeom Kim, Hyun Soo Kim, Jaeuk Hwang, Dahyun Yi, Jee Wook Kim

**Affiliations:** 1 Department of Neuropsychiatry, Hallym University Hangang Sacred Heart Hospital, Seoul, Republic of Korea; 2 Department of Psychiatry, Hallym University College of Medicine, Chuncheon, Gangwon, Republic of Korea; 3 Department of Neuropsychiatry, Hallym University Dongtan Sacred Heart Hospital, Hwaseong, Gyeonggi, Republic of Korea; 4 Department of Neuropsychiatry, Soonchunhyang University Bucheon Hospital, Bucheon, Republic of Korea; 5 Department of Laboratory Medicine, Hallym University Dongtan Sacred Heart Hospital, Hwaseong, Gyeonggi, Republic of Korea; 6 Department of Psychiatry, Soonchunhyang University Hospital, Seoul, Republic of Korea; 7 Institute of Human Behavioral Medicine, Medical Research Center Seoul National University, Seoul, Republic of Korea

**Keywords:** hopefulness, cognition, cognitively normal, Alzheimer’s disease, physical activity

## Abstract

**Background:**

With dementia cases rising globally, identifying modifiable psychological factors that support cognitive resilience is crucial. Hopefulness, an optimistic emotional state, may serve as a protective factor against cognitive decline. However, its role in cognitively normal (CN) individuals remains underexplored. We aim to investigate the association between self-reported hopefulness and overall cognitive function in CN older adults and to examine the moderating effect of physical activity on this relationship.

**Methods:**

A total of 152 CN adults aged 65–90 years were included in the General Lifestyle and Alzheimer’s Disease (AD) study. Hopefulness was assessed by the Geriatric Depression Scale item “Are you hopeful about the future?,” with “Yes” and “No” responses defining the hopefulness and non-hopefulness groups. Cognitive function was measured using the total score (TS) of the Consortium to Establish a Registry for Alzheimer’s Disease (CERAD) neuropsychological battery. Physical activity was evaluated using the Physical Activity Scale for the Elderly.

**Results:**

Participants in the hopefulness group showed significantly higher TS scores compared to the non-hopefulness group (B = 5.009, *p* = 0.003). Physical activity moderated this relationship, with a stronger positive association observed in individuals with high-to-moderate activity levels (B = 7.409, *p* < 0.001).

**Conclusion:**

Self-reported hopefulness, defined as optimism about the future, is positively associated with cognitive function in CN older adults, particularly among those with high-to-moderate physical activity levels. Interventions promoting both emotional well-being and physical activity may offer synergistic benefits for cognitive health.

## Introduction

With the rising prevalence of dementia and cognitive decline worldwide, identifying factors that may help prevent or delay these conditions has become increasingly important. Dementia, which affects over 55 million people globally, is projected to triple in prevalence by 2050 due to aging populations and lifestyle-related risks, underscoring the urgent need for effective prevention strategies (Livingston et al., 2020; Reuben et al., 2024). While much of the research to date has focused on genetic and biological risk factors, including apolipoprotein ε4 (APOE4) allele status and the pathophysiological roles of amyloid-beta and tau proteins (Kunkle et al., 2019; De Strooper and Karran, 2016; Scheltens et al., 2021), these factors alone do not fully account for individual differences in cognitive decline or resilience. Consequently, attention has turned to modifiable factors that may complement these biological findings and provide actionable pathways for intervention.

Recent studies have highlighted the potential of psychological factors, particularly emotional states, in influencing cognitive health. Emotional well-being, encompassing both the presence of positive emotions and the absence of chronic negative states, has been increasingly recognized as a determinant of cognitive trajectories in aging populations (Cohen et al., 2016; Lee et al., 2024; Kim et al., 2022). While the detrimental effects of negative thought patterns and chronic stress are well-documented—linking these factors to increased risks of cognitive decline and dementia through mechanisms such as hippocampal atrophy and neuroinflammation (Robertson et al., 2016; Saiz-Vazquez et al., 2021; Wallensten et al., 2023; Marchant et al., 2020)—there remains a significant gap in understanding the protective effects of positive emotions. Notably, repetitive negative thinking has been associated with increased deposition of amyloid-beta and tau, two hallmark pathologies of Alzheimer’s disease (AD), further solidifying the importance of addressing emotional health in cognitive aging research (Marchant et al., 2020).

Among positive psychological factors, hopefulness warrants particular attention. Traditionally, hopefulness has been conceptualized as a multifaceted construct encompassing an optimistic outlook toward the future and confidence in one’s ability to achieve desired goals (Bernardo and Ramos, 2024; Snyder, 2002). It has been associated with reduced stress, enhanced psychological well-being, and healthier lifestyle behaviors, such as physical activity and adherence to medical treatments—all of which are established contributors to cognitive health (Schweizer et al., 2019). Furthermore, its cognitive components, such as agency and pathways thinking, align closely with goal-directed behavior and problem-solving processes that support executive function and other domains of cognition (Snyder, 2002).

However, comprehensive assessment of such a multidimensional construct poses practical challenges in cohort studies of older adults, where long or complex questionnaires increase response burden and reduce data quality (Rolstad et al., 2011; Kutschar et al., 2019). For this reason, hopefulness was operationalized in the present study as self-reported optimism about the future, reflecting a concise, subjective perception of hope rather than the full theoretical construct. This operational definition allows for an investigation of how a simple sense of optimism relates to cognitive health in real-world aging populations. Recent studies in positive psychology have reinforced this association, showing that higher levels of optimism and hope predict slower cognitive decline and greater cognitive resilience among older adults (Marino et al., 2025; Liu et al., 2024; Vasileiou et al., 2025).

Cognitive decline itself may undermine positive emotional states, including hopefulness, by impairing the neural circuits involved in emotional regulation and future-oriented thinking. For example, age-related reductions in the functional connectivity of the prefrontal cortex and amygdala have been linked to diminished positive emotional reactivity (Schweizer et al., 2019). This bidirectional relationship underscores the importance of studying the role of hopefulness in cognitively normal individuals, where the potential for fostering and sustaining this emotional state remains intact. By exploring the relationship between hopefulness and cognitive function in this population, researchers may uncover novel insights into the mechanisms by which psychological factors influence brain health and identify new avenues for prevention. In addition to psychological factors, demographic, genetic, vascular, and lifestyle characteristics—such as age, sex, APOE4 genotype, vascular risk, and physical activity—have been shown to affect cognitive aging and may interact with emotional factors to shape cognitive outcomes (Carstensen et al., 2011; Gur and Gur, 2017; Niti et al., 2008). Given prior evidence that lifestyle factors can influence the strength of associations between emotional and cognitive health, we further explored whether physical activity moderates the relationship between self-reported hopefulness and cognitive function (Carstensen et al., 2011; Gur and Gur, 2017; Niti et al., 2008; Richman et al., 2005; Erickson et al., 2012; Engeroff et al., 2019).

This study aims to test the hypothesis that self-reported hopefulness is positively associated with better overall cognitive function, as measured by the total score (TS) of the Consortium to Establish a Registry for Alzheimer’s Disease (CERAD) assessment battery (Morris et al., 1989; Lee et al., 2002; Lee et al., 2004; Seo et al., 2010) in cognitively normal (CN) individuals. In addition, we examined whether the strength of this association differs depending on the level of physical activity, reflecting a potential interplay between psychological and behavioral factors in maintaining cognitive health. The findings from this study could enhance our understanding of how self-reported hopefulness contributes to cognitive resilience and inform strategies for promoting healthy cognitive aging.

## Materials and methods

### Participants

This study is part of the General Lifestyle and AD (GLAD) study, an ongoing prospective cohort study initiated in 2020 (Keum et al., 2024; Choe et al., 2023; Kim et al., 2023). As of September 2022, a total of 152 CN adults aged 65 to 90 years had been enrolled. Given that this study was part of the ongoing GLAD cohort, the sample size reflected all eligible participants who had completed full assessments at that time rather than a predefined target.

Participants were recruited through two complementary approaches. First, CN individuals who attended a dementia screening program at the memory clinic of Hallym University Dongtan Sacred Heart Hospital in Hwaseong, South Korea were invited for eligibility assessment. Second, additional CN volunteers were recruited from the local community through referrals by existing participants, family members, or acquaintances. These community-based participants were selected to ensure diversity in demographic characteristics and cognitive status, with the goal of improving the epidemiological representativeness of the study sample among older Korean adults. To minimize potential bias from recruitment source, we compared key demographic and cognitive variables between hospital- and community-based participants and found no significant differences (all *p* > 0.10). A binary variable indicating recruitment source was also included as a covariate in the regression analyses, and the main results remained unchanged.

To ensure cognitive normality, participants were required to have a Clinical Dementia Rating (CDR; Morris, 1993) score of 0 and no diagnosis of mild cognitive impairment (MCI) or dementia. Exclusion criteria included: (1) the presence of comorbid medical, psychiatric, or neurological conditions that could affect cognitive functioning; (2) severe communication or behavioral difficulties that could hinder clinical assessments; and (3) simultaneous participation in another clinical trial involving an investigational product.

The study protocol received approval from the Institutional Review Board of Hallym University Dongtan Sacred Heart Hospital and was carried out in compliance with the latest guidelines of the Declaration of Helsinki. Informed consent was obtained from all participants or their legal representatives.

### Clinical assessments

All participants underwent standardized clinical evaluations conducted by trained psychiatrists in accordance with the clinical assessment protocol of the GLAD study. This protocol incorporated the CERAD-K (Morris et al., 1989; Lee et al., 2002). Furthermore, trained neuropsychologists administered the GLAD neuropsychological assessment protocol, which included the CERAD-K neuropsychological battery (Lee et al., 2004). To explore the association between hope and global cognitive function, TS of the CERAD-K neuropsychological battery was utilized (Seo et al., 2010). The TS, representing an aggregate measure of overall cognitive performance, was calculated by summing the scores of seven subtests: verbal fluency, the modified Boston Naming Test, word list memory, constructional praxis, word list recall, word list recognition, and constructional recall.

Hopefulness was assessed using the fifth item of the 30-item Geriatric Depression Scale (GDS; Yesavage et al., 1982; Kim et al., 2008), which asks, “Are you hopeful about the future?” Participants answered “Yes” or “No.” Those who answered “Yes” were classified as the hopefulness group, and those who answered “No” as the non-hopefulness group. This item was chosen to capture participants’ self-perceived optimism about the future. Although it represents a single dichotomous measure, prior studies have shown that individual GDS items—particularly those assessing positive mood or hopelessness—have acceptable psychometric validity and are widely used as brief indicators of affective states in older adults (Kim et al., 2008; Chan, 1996; Huang et al., 2017; Kim et al., 2021). Based on this rationale, the construct was operationalized as self-reported hopefulness, reflecting each participant’s subjective outlook toward their future.

The severity of depressive symptoms was evaluated using the 30-item GDS (Yesavage et al., 1982; Kim et al., 2008), a widely validated tool for assessing depressive symptoms in older adults. Based on their GDS scores, participants were categorized into two groups: non-depressed (GDS score of 0–15) and major depressed (GDS score of ≥16; Bae and Cho, 2004). This classification facilitated the analysis of depressive symptom severity as a potential factor influencing cognitive function and emotional states, including hopefulness. Vascular risk factors (VRFs), including hypertension, diabetes mellitus, dyslipidemia, coronary heart disease, transient ischemic attack, and stroke, were evaluated through structured interviews with participants and their family members. Data collection was carried out by trained researchers. The vascular risk score (VRS) was derived by calculating the proportion of identified VRFs relative to the total number of assessed factors (DeCarli et al., 2004). Body mass index (BMI) was determined by dividing body weight in kilograms by the square of height in meters, following the guidelines established by the World Health Organization (WHO). Detailed criteria for BMI computation can be found on the WHO’s official website^1^ (Kim et al., 2022). Physical activity levels were measured using the Korean version of the Physical Activity Scale for the Elderly (PASE; Choe et al., 2010; Washburn et al., 1993; Lee et al., 2023). The PASE score was computed by summing weighted scores from three activity domains: leisure, household, and occupational activities. Higher PASE scores reflect greater overall physical activity. Participants were initially divided into tertiles according to total PASE scores (low, moderate, and high). Further analytic details regarding the merging of categories based on statistical similarity are described in the Results section. Participants were stratified into three income categories: (1) below the minimum cost of living (MCL), (2) between the MCL and twice the MCL, and (3) at least twice the MCL. These classifications were based on standards defined by the Ministry of Health and Welfare, Republic of Korea^2^ (Kim et al., 2020; Kim et al., 2021). Lifetime alcohol consumption (categorized as never, former, or current drinker) and smoking history (categorized as never, ex-smoker, or current smoker) were determined through structured interviews conducted by trained researchers and supplemented by medical record reviews (Kim et al., 2022). Dietary patterns were comprehensively evaluated using the Mini Nutritional Assessment (MNA; Vellas et al., 1999), a validated and widely used tool for assessing the nutritional status of older adults. This assessment involved detailed interviews focusing on participants’ typical dietary habits and specific food types consumed regularly. Key food groups analyzed included high-quality protein sources, fresh fruits, and a variety of vegetables (Vellas et al., 1999).

Blood samples were collected via venipuncture in the morning (8–9 a.m.) after participants had fasted overnight. Hemoglobin levels were measured using the XN-3000 automated hematologic analyzer and its dedicated reagents (Sysmex, Kobe, Japan), ensuring precision in the evaluation of anemia and overall hematologic status. Biochemical parameters, including albumin, glucose, and lipid profiles (high-density lipoprotein [HDL] cholesterol and low-density lipoprotein [LDL] cholesterol), were analyzed using the COBAS c702 analyzer with dedicated reagents (Roche Diagnostics, Mannheim, Germany). APOE genotyping was performed using the APOE ACE Genotyping Kit (Seegene, Seoul, Korea). APOE4 positivity, indicative of an increased genetic risk for AD, was defined as the presence of at least one ε4 allele.

### Statistical analyses

To compare continuous variables, including demographic and clinical characteristics, two-tailed independent *t*-tests were employed, while categorical data were analyzed using chi-square tests. To investigate the association between self-reported hopefulness and overall cognitive function, multiple linear regression analyses were conducted, with self-reported hopefulness as the independent variable and overall cognitive function as the dependent variable. The non-hopefulness group served as the reference category.

A two-step modeling approach was applied to progressively adjust for covariates. The first model excluded all covariates, while the second model included adjustments for age, sex, APOE4 status, VRS, BMI, annual income, PASE total score, alcohol intake, smoking, dietary habits, and blood nutritional markers. To disentangle the effect of self-reported hopefulness from general depressive symptoms, the total GDS score excluding the fifth item assessing hopefulness was also included as a covariate in all regression models. Additionally, to further evaluate the incremental contribution of self-reported hopefulness beyond these covariates, hierarchical multiple regression analyses were performed. In this analysis, all control variables were entered in the first block (Model 1), and self-reported hopefulness was added in the second block (Model 2) to assess the change in explained variance (ΔR; Reuben et al., 2024).

Potential moderating effects of covariates (age, sex, APOE4 status, VRS, BMI, and PASE total score) were evaluated by incorporating two-way interaction terms into the regression models. These variables were selected based on prior literature showing that demographic, genetic, and lifestyle factors can modify the association between emotional factors and cognitive outcomes (Carstensen et al., 2011; Gur and Gur, 2017; Niti et al., 2008). When significant interactions were identified, stratified linear regression analyses were subsequently performed to further elucidate these relationships.

Sensitivity analyses were carried out to minimize confounding effects related to physical or mental health conditions. Specifically, analyses were repeated after excluding participants with self-reported major depression. This step aimed to isolate the relationship between self-reported hopefulness and overall cognitive function from the potential influence of depression. All statistical analyses were conducted using IBM SPSS Statistics software (version 28.0; IBM Corp, Armonk, NY, United States).

## Results

### Participants characteristics

The demographic and clinical characteristics of the study participants, stratified by self-reported hopefulness status, are presented in Table 1. Of the 152 participants, 77 were categorized as belonging to the self-reported hopefulness group, while 75 were assigned to the non-hopefulness group. Participants in the hopefulness group demonstrated superior performance on overall cognition and exhibited lower levels of depression. Importantly, none of the participants were classified as malnourished, defined as having serum albumin levels below 3.5 g/dL (Cabrerizo et al., 2015).

**Table 1:** Participant characteristics according to self-reported hopefulness status.

Characteristic	Overall	Hopefulness	Non-hopefulness	*p*
n	152	77	75	
Age, y	72.15 (5.57)	71.59 (5.68)	72.72 (5.42)	0.215^a^
Female, n (%)	108 (71.05)	55 (71.43)	53 (70.67)	0.918^b^
Education, y	9.76 (4.57)	10.43 (4.50)	9.07 (4.57)	0.066^a^
MMSE score	26.29 (2.89)	26.70 (2.80)	25.87 (2.95)	0.075^a^
APOE4-positivity, n (%)	22 (14.47)	11 (14.43)	11 (14.67)	0.947^b^
VRS, %	24.23 (18.28)	24.03 (17.83)	24.44 (18.85)	0.888^a^
BMI	24.72 (3.06)	24.71 (2.45)	24.73 (3.60)	0.965^a^
CN, n (%)	152 (100.00)	77 (100.00)	75 (100.00)	
Annual income, n (%)				0.127^b^
<MCL	15 (9.87)	7 (9.09)	8 (10.67)	
≥ MCL, <2 × MCL	44 (28.95)	17 (22.08)	27 (36.00)	
≥ 2 × MCL	93 (61.18)	53 (68.83)	40 (53.33)	
PASE total score	68.75 (48.41)	68.00 (46.67)	69.53 (50.44)	0.846^a^
PASE, n (%)				0.916^b^
High-to-moderate	103 (67.76)	51 (66.23)	52 (69.33)	
Low	49 (32.24)	26 (33.77)	23 (30.67)	
Alcohol drink status, n (%)				0.436^b^
Never	85 (55.92)	47 (61.04)	38 (50.67)	
Former	27 (17.76)	12 (15.58)	15 (20.00)	
Drinker	40 (26.32)	18 (23.38)	22 (29.33)	
Smoking status, n (%)				0.937^c^
Never	121 (79.61)	62 (80.52)	59 (78.67)	
Former	27 (17.76)	13 (16.88)	14 (18.67)	
Smoker	4 (2.63)	2 (2.60)	2 (2.67)	
Blood nutritional markers				
Albumin, g/dL	4.59 (0.27)	4.59 (0.27)	4.60 (0.27)	0.792^a^
Glucose, fasting, mg/dL	108.85 (20.84)	109.61 (20.98)	108.05 (20.81)	0.648^a^
HDL-cholesterol, mg/dL	55.91 (13.02)	56.53 (13.45)	55.27 (32.40)	0.553^a^
LDL-cholesterol, mg/dL	94.82 (31.77)	95.35 (31.35)	94.27 (32.40)	0.835^a^
Nutritional markers				
Protein, No (%)				0.841^b^
High	73 (48.03)	36 (46.75)	37 (49.33)	
Moderate	54 (35.53)	27 (35.06)	27 (36.00)	
Low	25 (16.45)	14 (18.18)	11 (14.47)	
Fruit & Vegetables, No (%)				0.336^b^
High	59 (38.82)	27 (35.06)	32 (42.67)	
Low	93 (61.18)	50 (64.94)	43 (57.33)	
GDS total score	10.80 (7.20)	8.22 (6.21)	13.45 (7.22)	<0.001^a^
GDS, n (%)				0.034^b^
None (<16)	117 (76.97)	65 (84.42)	52 (69.33)	
Major depressed (≥16)	35 (23.03)	12 (15.58)	23 (30.67)	
CERAD cognition				
Total score	74.57 (12.66)	77.96 (12.50)	71.08 (11.93)	<0.001^a^

MMSE, mini-mental state examination; APOE4, apolipoprotein E ε4 allele; VRS, vascular risk score; BMI, body mass index; CN, cognitively normal; MCL, minimum cost of living; PASE, physical activity scale for the elderly; HDL, high density lipoprotein; LDL, low density lipoprotein; GDS, geriatric depression scale; CERAD, Consortium to Establish a Registry for Alzheimer’s Disease.

Data are expressed as mean (standard deviation), unless otherwise indicated.

a By one-way analysis of variance.

b By chi-square test.

c By fisher exact test.

### Association between self-reported hopefulness and cognition

**Figure 1:**
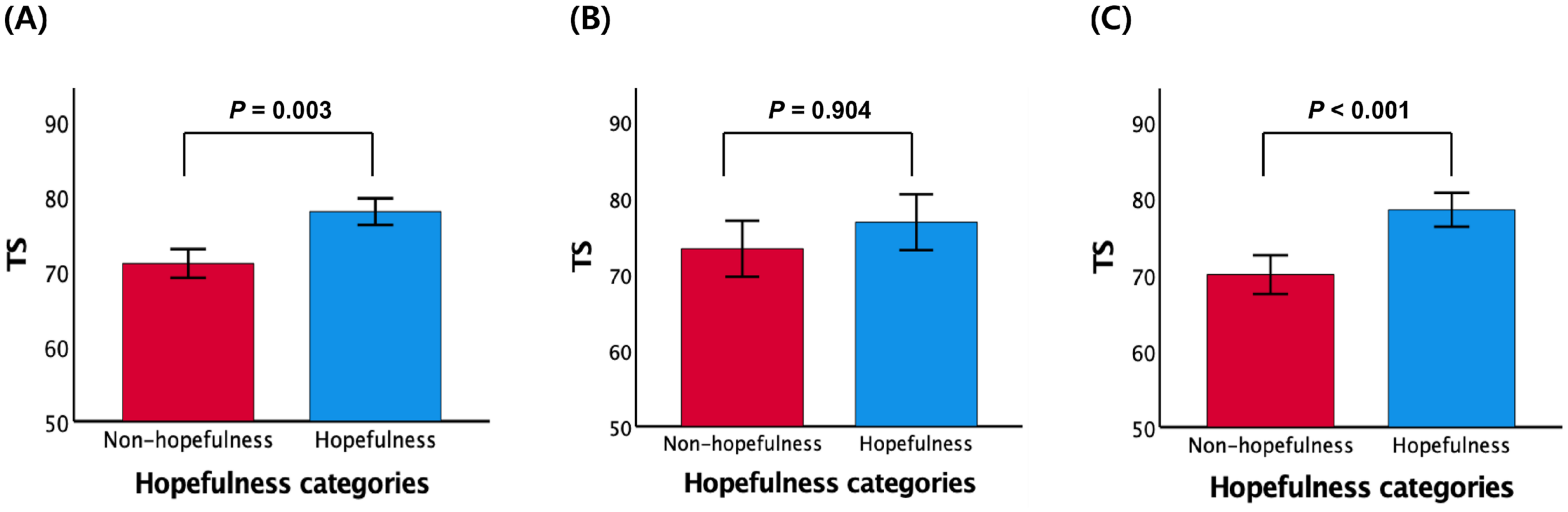
Plots of the associations between self-reported hopefulness (defined by responses to the GDS item “Are you hopeful about the future?”) and overall cognition **(A–C)**: **(A)** hopefulness categories vs. total score (TS), **(B)** hopefulness categories vs. TS among participants with high-to-moderate physical activity, and **(C)** hopefulness categories vs. TS among participants with low physical activity. **(A–C)** were adjusted for potential covariates; mean cognition values are presented and error bars represent standard error.

Participants in the self-reported hopefulness group exhibited significantly higher overall cognition compared to those in the non-hopefulness group, even after adjusting for potential confounders (B = 5.009, SE = 1.667, *β* = 0.198, *p* = 0.003; Table 2; Figure 1A; Supplementary Table S1). This result remained consistent after further adjustment for the total GDS score excluding the hopefulness item (B = 4.481, SE = 1.737, *β* = 0.177, *p* = 0.011; Supplementary Table S2). Hierarchical regression analysis further showed that adding hopefulness to the model with all control variables significantly improved the explained variance in cognition (ΔR^2^ = 0.036, *p* = 0.003), indicating a small-to-medium effect size (Supplementary Table S3).

**Table 2:** Results of multiple linear regression analyses of the associations between self-reported hopefulness and overall cognitive function (N = 152).

	Total score of CERAD			
	B	SE	β	*p*
Model 1^a^				
Hopefulness	6.907	1.996	0.273	<0.001
Non-hopefulness		Reference		
Model 2^b^				
Hopefulness	5.009	1.667	0.198	0.003
Non-hopefulness		Reference		

CERAD, Consortium to Establish a Registry for Alzheimer’s Disease; APOE4, apolipoprotein ε4; VRS, vascular risk score; BMI, body mass index; HDL, high density lipoprotein; LDL, low density lipoprotein.

a Crude model (no adjustment for covariates).

b Model adjusted for age, sex, education, APOE4, VRS, PASE total score, BMI, annual income, alcohol intake, smoking, dietary habits (protein, fruit and vegetable intake), and blood-based nutritional markers (albumin, glucose, HDL, and LDL cholesterol).

### Moderation of the association between self-reported hopefulness and cognition

A significant interaction was identified between self-reported hopefulness and physical activity (B = 0.081, SE = 0.033, *β* = 0.526, *p* = 0.014), suggesting that physical activity moderates the relationship between self-reported hopefulness and overall cognitive function (Table 3). Conversely, interactions between self-reported hopefulness and other variables, including age, sex, APOE4 status, VRS, and BMI, remained non-significant (Table 3).

**Table 3:** Results of multiple linear regression analyses including self-reported hopefulness × one covariate interaction term, predicting overall cognitive function.

	Total score of CERAD			
	B	SE	β	*p*
Hopefulness	10.930	5.061	0.432	0.033
Age	2.225	5.729	0.081	0.698
Hopefulness × Age	−4.460	3.601	−0.337	0.218
Hopefulness	6.007	2.007	0.237	0.003
Sex	1.140	6.303	0.041	0.857
Hopefulness × Sex	−3.296	3.684	−0.191	0.373
Hopefulness	5.448	1.799	0.215	0.003
APOE4	3.319	7.662	0.091	0.666
Hopefulness × APOE4	−3.109	4.731	−0.137	0.512
Hopefulness	7.699	2.895	0.304	0.009
VRS	0.220	0.152	0.316	0.150
Hopefulness × VRS	−0.106	0.094	−0.259	0.258
Hopefulness	1.872	14.618	0.074	0.898
BMI	0.499	0.849	0.119	0.557
Hopefulness × BMI	0.127	0.586	0.130	0.829
Hopefulness	−0.915	2.703	−0.036	0.736
PASE	−0.093	0.050	−0.354	0.067
Hopefulness × PASE	0.081	0.033	0.526	0.014

CERAD, Consortium to Establish a Registry for Alzheimer’s Disease; APOE4, apolipoprotein ε4; VRS, vascular risk score; BMI, body mass index; HDL, high density lipoprotein; LDL, low density lipoprotein.Model adjusted for age, sex, education, APOE4, VRS, PASE total score, BMI, annual income, alcohol intake, smoking, dietary habits (protein, fruit and vegetable intake), and blood-based nutritional markers (albumin, glucose, HDL, and LDL cholesterol).

To clarify this interaction, stratified regression analyses were conducted based on tertiles of PASE total scores. Both the high and moderate activity groups showed comparable positive associations between self-reported hopefulness and overall cognitive function, whereas the low activity group showed no significant relationship (Supplementary Table S4). Given these similar patterns, the high and moderate groups were combined into a single high-to-moderate activity category for the main analysis. In this combined group, self-reported hopefulness was significantly associated with higher overall cognitive function (B = 7.409, SE = 1.998, *β* = 0.288, *p* < 0.001), whereas the association remained non-significant in the low activity group (B = 0.444, SE = 3.641, *β* = 0.018, *p* = 0.904; Table 4; Figures 1B,C).

**Table 4:** Results of multiple linear regression analyses of the associations between self-reported hopefulness and overall cognitive function according to physical activity status (*N* = 152).

	Total score of CERAD			
	B	SE	β	*p*
Low physical activity (*n* = 49)				
Model 1^a^				
Hopefulness	3.498	3.515	0.144	0.325
Non-hopefulness	Reference			
Model 2^b^				
Hopefulness	0.444	3.641	0.018	0.904
Non-hopefulness	Reference			
High-to-moderate physical activity (*n* = 103)				
Model 1^a^				
Hopefulness	8.510	2.432	0.330	<0.001
Non-hopefulness	Reference			
Model 2^b^				
Hopefulness	7.409	1.998	0.288	<0.001
Non-hopefulness	Reference			

CERAD, Consortium to Establish a Registry for Alzheimer’s Disease; APOE4, apolipoprotein ε4; VRS, vascular risk score; BMI, body mass index; HDL, high density lipoprotein; LDL, low density lipoprotein.

a Crude model (no adjustment for covariates).

b Model adjusted for age, sex, education, APOE4, VRS, BMI, annual income, alcohol intake, smoking, dietary habits (protein, fruit and vegetable intake), and blood-based nutritional markers (albumin, glucose, HDL, and LDL cholesterol).

### Sensitivity analyses

Older individuals without self-reported major depression exhibited overall cognitive performance comparable to that of the overall cohort (Supplementary Tables S5, S6).

## Discussion

This study investigated the association between self-reported hopefulness and overall cognitive function in CN older adults. Results showed that participants in the self-reported hopefulness group had significantly better cognitive performance compared to the non-hopefulness group. Importantly, physical activity moderated this relationship: the positive association between self-reported hopefulness and cognition was observed only among individuals with high-to-moderate levels of physical activity, while no significant association was found among those with low activity. Sensitivity analyses confirmed the robustness of these findings, even after excluding participants with self-reported major depression.

The present study builds on existing evidence emphasizing the role of positive psychological factors in cognitive health while providing novel insights into the specific relationship between self-reported hopefulness and cognitive function. Prior studies have consistently demonstrated that positive emotional states contribute to cognitive resilience through pathways such as stress reduction, improved emotional regulation, and healthier lifestyle behaviors (Steptoe et al., 2015; Snyder et al., 1991). For instance, subjective well-being, which encompasses positive emotions, is associated with slower cognitive decline and reduced dementia risk in older adults (Steptoe et al., 2015). Additionally, hopefulness has been conceptualized as a cognitive process involving goal-directed determination and pathways thinking, which together support problem-solving and executive function, key components of cognitive reserve (Snyder et al., 1991). In contrast, the detrimental effects of negative psychological states have been widely reported (Marchant and Howard, 2015; Lupien et al., 2009) Repetitive negative thinking has been shown to accelerate cognitive decline by increasing amyloid-beta and tau deposition, the hallmark pathologies of AD (Marchant and Howard, 2015). Similarly, chronic stress has been linked to hippocampal atrophy and impaired cognitive performance due to dysregulation of the hypothalamic–pituitary–adrenal (HPA) axis and elevated cortisol levels (Lupien et al., 2009). While these studies underscore the importance of emotional health in cognitive aging, the current study uniquely focuses on hopefulness, a forward-looking positive emotional state, and its association with overall cognitive function.

Another key finding of this study is the moderating role of physical activity in the relationship between self-reported hopefulness and overall cognitive function. In the fully adjusted model, self-reported hopefulness was positively associated with cognitive function. When the interaction term was added, the conditional main effect of hopefulness—evaluated at zero physical activity—was not significant, reflecting its effect under the specific condition of no physical activity rather than an absence of overall association. The interaction between self-reported hopefulness and physical activity remained positive and significant, indicating that the cognitive benefits of hopefulness become stronger as physical activity increases. In other words, the cognitive benefits of self-reported hopefulness become more pronounced among those who engage in regular physical activity, reflecting a synergistic rather than contradictory relationship. These results are consistent with prior evidence that physical activity enhances neuroplasticity, increases brain-derived neurotrophic factor (BDNF) expression, and improves emotion regulation, thereby reinforcing the positive influence of self-reported hopefulness on cognitive outcomes (Cefis et al., 2023; Gomutbutra et al., 2020; Miranda et al., 2019; Gao et al., 2022; Phillips, 2017; Zoladz and Pilc, 2010; Voss et al., 2013; Stillman et al., 2016; Norouzi et al., 2019).

Interestingly, unlike previous research suggesting that genetic and vascular risks, such as APOE4 allele status and VRS, amplify the risk of cognitive decline (Corder et al., 1993; Jack et al., 2018), the current study did not observe moderating effects of these variables. For example, APOE4 carriers exhibit increased vulnerability to cognitive deficits, even in cognitively normal individuals (Corder et al., 1993; Jack et al., 2018). However, in our study, the lack of moderation could be attributed to the homogeneity of the sample, which exclusively included CN older adults. The effects of APOE4 and other risk factors may not yet manifest in this group due to their preserved cognitive status. Additionally, limited variability in age and vascular risk within the sample may have reduced their moderating potential.

Taken together, while prior research has explored the impact of positive and negative emotional states on cognition, as well as the independent effects of physical activity and genetic risk factors, the present study extends prior work by showing that the association between self-reported hopefulness and cognition is conditional on physical activity level, with the effect being stronger among individuals with high-to-moderate levels of physical activity. This novel finding underscores the importance of examining psychological and physiological factors together, rather than in isolation, to fully understand their contributions to cognitive health. By focusing specifically on self-reported hopefulness in a CN population, this study advances our understanding of how modifiable emotional and behavioral factors can be harnessed to promote cognitive resilience and delay cognitive decline.

The findings of this study underscore the importance of fostering self-reported hopefulness as a potential strategy for cognitive preservation in older adults. Interventions designed to promote positive emotions, such as cognitive-behavioral therapy, mindfulness-based stress reduction, and future-oriented goal-setting programs, may hold promise for supporting cognitive health. Moreover, the observed moderating role of physical activity suggests that multi-component interventions combining psychological and physical strategies may yield the greatest benefits. Encouraging older adults to engage in regular physical activity while fostering self-reported hopefulness could represent a practical and cost-effective approach to delaying cognitive decline.

Several mechanisms may explain the relationship between self-reported hopefulness and cognitive function. First, hopefulness may reduce stress by stabilizing the HPA axis and lowering cortisol levels, mitigating hippocampal atrophy and cognitive impairment (Lupien et al., 2009; Juster et al., 2010). Second, hopefulness strengthens cognitive reserve through goal-directed thinking and problem-solving, key components of executive function, delaying cognitive decline (Snyder et al., 1991). In addition, hopefulness is linked to healthier lifestyle behaviors, such as regular physical activity, balanced diet, and medical adherence, which collectively contribute to cognitive preservation (Stern, 2012; Schiavon et al., 2016). Recent findings from positive psychology research also support these mechanisms, suggesting that interventions enhancing hope and optimism—such as cognitive-behavioral and mindfulness-based approaches—can improve emotional regulation, neuroplasticity, and cognitive performance in both healthy and at-risk older adults (Marino et al., 2025; Liu et al., 2024; Vasileiou et al., 2025). Finally, both hopefulness and physical activity may converge on neuroplastic mechanisms. Positive psychological states and physical exercise are known to increase brain-derived neurotrophic factor (BDNF) levels and improve prefrontal–limbic connectivity, fostering synaptic plasticity and emotional regulation (Gomutbutra et al., 2020; Miranda et al., 2019; Gao et al., 2022; Phillips, 2017; Zoladz and Pilc, 2010; Voss et al., 2013; Stillman et al., 2016; Norouzi et al., 2019). Taken together, these complementary pathways suggest that physical activity may amplify the neurobiological and behavioral benefits of hopefulness, jointly promoting cognitive resilience and healthy brain aging (Dinas et al., 2011; Jakicic et al., 2019).

## Strengths and limitations

This study systematically investigated the association between self-reported hopefulness and overall cognitive function in a well-characterized cohort of CN older adults. The analysis rigorously adjusted for a comprehensive range of confounding variables, including demographic, clinical, and lifestyle factors, ensuring the robustness and reliability of the findings. Furthermore, the identification of the moderating role of physical activity provides novel insights into the interplay between psychological and physiological factors in maintaining cognitive health, highlighting a potential avenue for targeted interventions. The study has several limitations. First, self-reported hopefulness was assessed using a single item from the GDS, which, although pragmatic and commonly used, may not fully capture the multidimensional nature of hope, such as agency and pathways thinking. Future studies should incorporate validated and multidimensional instruments, such as the Snyder Hope Scale (Snyder et al., 1991), to better characterize this construct and its neural and behavioral correlates. Second, the cross-sectional design prevents causal inferences about the relationship between self-reported hopefulness and cognitive function, necessitating longitudinal research. Third, the positive association observed may be influenced by other depressive symptoms. Additional analyses controlling for the total GDS score (excluding the hopefulness item) and removing participants with major depressive disorder yielded consistent findings, minimizing this concern. Fourth, cognitive decline linked to mild cognitive impairment or dementia could reduce self-reported hopefulness. To address this, only CN individuals were included, and analyses excluding participants with severe depression confirmed the results. Finally, although participants were recruited from both hospital- and community-based sources to enhance demographic diversity, all recruitment and assessments were conducted within a single research center. Therefore, while the sample included individuals with varying backgrounds, the single-center design may limit the generalizability of the findings. Multi-center studies with larger and more diverse populations are warranted to confirm these results.

In conclusion, our study demonstrated that self-reported hopefulness is positively associated with cognitive function in CN older adults, particularly when paired with high-to-moderate physical activity. These findings support the development of interventions targeting both psychological and physical health to promote cognitive resilience and prevent dementia.

## Data Availability

The datasets are not publicly available due to Institutional Review Board restrictions for privacy protection. Data may be accessed upon reasonable request and IRB approval by contacting yoon4645@gmail.com.

## Footnotes

^1^ https://www.euro.who.int/en/health-topics/disease-prevention/nutrition/a-healthy-lifestyle/body-mass-index-bmi

^2^ http://www.law.go.kr
