# Supplementary material for: Self-reported hopefulness and cognitive function: the moderating effect of physical activity in older adults without cognitive impairment
Source: Front Aging Neurosci. 2025 Nov 14;17:1646298. doi: 10.3389/fnagi.2025.1646298 (PMC12660227; doi:10.3389/fnagi.2025.1646298)
Supplement: Supplementary file 1 [file Data_Sheet_1.docx]

| **Table S1.** Results of multiple linear regression analyses of the associations between self-reported hopefulness and overall cognitive function (N = 152) | | | | | | | | |
| --- | --- | --- | --- | --- | --- | --- | --- | --- |
|  |  |  | Total score of CERAD | | | | |  |
|  |  |  | B | SE | $\beta$ | | *p* |  |
| Model 1 ^a^ |  |  |  |  | |  |  |  |
| Hopefulness |  |  | 6.907 | 1.996 | | 0.273 | <0.001 |  |
| Model 2 ^b^ |  |  |  |  | |  |  |  |
| Hopefulness |  |  | 5.009 | 1.667 | | 0.198 | 0.003 |  |
| Age |  |  | -4.486 | 1.866 | | -0.163 | 0.018 |  |
| Sex |  |  | -3.934 | 2.746 | | -0.141 | 0.154 |  |
| Education |  |  | 8.595 | 1.376 | | 0.494 | <0.001 |  |
| APOE4 |  |  | -1.440 | 2.498 | | -0.039 | 0.565 |  |
| VRS |  |  | 0.059 | 0.055 | | 0.085 | 0.287 |  |
| PASE total score |  |  | 0.029 | 0.017 | | 0.109 | 0.100 |  |
| BMI |  |  | 0.184 | 0.173 | | 0.081 | 0.289 |  |
| Annual income |  |  | 0.878 | 1.397 | | 0.046 | 0.531 |  |
| Alcohol intake |  |  | 1.108 | 1.073 | | 0.075 | 0.304 |  |
| Smoking |  |  | 1.888 | 2.557 | | 0.072 | 0.461 |  |
| Protein intake |  |  | 1.380 | 0.714 | | 0.131 | 0.055 |  |
| Fruit and vegetable intake |  |  | 0.830 | 1.937 | | 0.030 | 0.669 |  |
| Albumin |  |  | 1.027 | 3.174 | | 0.022 | 0.461 |  |
| Glucose |  |  | -0.068 | 0.042 | | -0.111 | 0.112 |  |
| HDL-cholesterol |  |  | 0.022 | 0.068 | | 0.023 | 0.743 |  |
| LDL-cholesterol |  |  | 0.027 | 0.030 | | 0.067 | 0.370 |  |
| CERAD Consortium to Establish a Registry for Alzheimer’s Disease, APOE4 apolipoprotein ε4, VRS vascular risk score, BMI body mass index, PASE physical activity scale for the elderly, HDL high density lipoprotein, LDL low density lipoprotein.  ^a^ Crude model (no adjustment for covariates).  ^b^ Model adjusted for age, sex, education, APOE4, VRS, PASE total score, BMI, annual income, alcohol intake, smoking, dietary habits (protein, fruit and vegetable intake), and blood-based nutritional markers (albumin, glucose, HDL, and LDL cholesterol). | | | | | | | | |

| **Table S2** Results of multiple linear regression analyses of the associations between self-reported hopefulness and overall cognitive function, adjusted for total GDS scores excluding the hopefulness item (N = 152) | | | | | | | |
| --- | --- | --- | --- | --- | --- | --- | --- |
|  |  |  | Total score of CERAD | | | | |
|  |  |  | B | SE | $\beta$ | | *p* |
| Model 1 ^a^ |  |  |  |  | |  |  |
| Hopefulness |  |  | 4.481 | 1.737 | | 0.177 | 0.011 |
| Non-hopefulness |  |  |  | Reference | |  |  |
| CERAD Consortium to Establish a Registry for Alzheimer’s Disease, APOE4 apolipoprotein ε4, VRS vascular risk score, BMI body mass index, PASE physical activity scale for the elderly, HDL high density lipoprotein, LDL low density lipoprotein.  ^a^ Model adjusted for age, sex, education, APOE4, VRS, PASE total score, BMI, annual income, alcohol intake, smoking, dietary habits (protein, fruit and vegetable intake), blood-based nutritional markers (albumin, glucose, HDL, and LDL cholesterol), and total GDS scores excluding the hopefulness item. | | | | | | | |

| **Table S3.** Results of hierarchical multiple linear regression analyses of the associations between self-reported hopefulness and overall cognitive function (N = 152) | | | | | | |
| --- | --- | --- | --- | --- | --- | --- |
| Model | Variables included |  | R² | Adjusted R² | ΔR² | *P value*  *for* ΔR² |
| Model 1 | Age, sex, education, APOE4, VRS, PASE total score, BMI, annual income, alcohol intake, smoking, dietary habits (protein, fruit and vegetable intake), and blood-based nutritional markers (albumin, glucose, HDL, and LDL cholesterol). |  | 0.432 | 0.365 | - | - |
| Model 2 | Model 1+ Hopefulness |  | 0.468 | 0.401 | 0.036 | 0.003 |
| Model 1 included 16 control variables; Model 2 added hopefulness. ΔR² = R²(Model 2) – R²(Model 1).  According to Cohen (1988), ΔR² = 0.036 corresponds to a small-to-medium effect size, indicating a significant incremental contribution of  hopefulness (p = 0.003). | | | | | | |
| APOE4 apolipoprotein ε4, VRS vascular risk score, BMI body mass index, PASE physical activity scale for the elderly, HDL high density  lipoprotein, LDL low density lipoprotein. | | | | | | |

| **Table S4.** Results of multiple linear regression analyses of the associations between self-reported hopefulness and overall cognitive function across high and moderate physical activity groups (N = 103) | | | | | | |  |
| --- | --- | --- | --- | --- | --- | --- | --- |
|  |  |  | Total score of CERAD | | | | |
|  |  |  | B | SE | $\beta$ | *p* | |
| *Moderate physical activity (n = 53)* | | | | | | | |
| Model 1 ^a^ |  |  |  | | |  | |
| Hopefulness |  |  | 7.788 | 2.994 | 0.293 | 0.011 | |
| Non-hopefulness |  |  | Reference | | |  | |
| Model 2 ^b^ |  |  |  | | |  | |
| Hopefulness |  |  | 5.922 | 2.690 | 0.223 | 0.032 | |
| Non-hopefulness |  |  | Reference | | |  | |
| *High physical activity (n = 50)* | | | | | | | |
| Model 1 ^a^ |  |  |  | | |  | |
| Hopefulness |  |  | 11.245 | 3.531 | 0.421 | 0.003 | |
| Non-hopefulness |  |  | Reference | | |  | |
| Model 2 ^b^ |  |  |  | | |  | |
| Hopefulness |  |  | 10.063 | 3.676 | 0.377 | 0.010 | |
| Non-hopefulness |  |  | Reference | | |  | |
| CERAD Consortium to Establish a Registry for Alzheimer’s Disease, APOE4 apolipoprotein ε4, VRS vascular risk score, BMI body mass index, HDL high density lipoprotein, LDL low density lipoprotein.  ^a^ Crude model (no adjustment for covariates).  ^b^ Model adjusted for age, sex, education, APOE4, VRS, BMI, annual income, alcohol intake, smoking, dietary habits (protein, fruit and vegetable intake), and blood-based nutritional markers (albumin, glucose, HDL, and LDL cholesterol). | | | | | | |  |

| **Table S5** Results of multiple linear regression analyses of the associations between self-reported hopefulness and overall cognitive function in older adults without major depression (N = 117) | | | | | | | | |
| --- | --- | --- | --- | --- | --- | --- | --- | --- |
|  |  |  | Total score of CERAD | | | | |  |
|  |  |  | B | SE | $\beta$ | | *p* |  |
| Model 1 ^a^ |  |  |  |  | |  |  |  |
| Hopefulness |  |  | 6.929 | 2.375 | | 0.264 | 0.004 |  |
| Non-hopefulness |  |  |  | Reference | |  |  |  |
| Model 2 ^b^ |  |  |  |  | |  |  |  |
| Hopefulness |  |  | 4.780 | 1.887 | | 0.182 | 0.013 |  |
| Non-hopefulness |  |  |  | Reference | |  |  |  |
| CERAD Consortium to Establish a Registry for Alzheimer’s Disease, APOE4 apolipoprotein ε4, VRS vascular risk score, BMI body mass index, PASE physical activity scale for the elderly, HDL high density lipoprotein, LDL low density lipoprotein.  ^a^ Crude model (no adjustment for covariates).  ^b^ Model adjusted for age, sex, education, APOE4, VRS, PASE total score, BMI, annual income, alcohol intake, smoking, dietary habits (protein, fruit and vegetable intake), and blood-based nutritional markers (albumin, glucose, HDL, and LDL cholesterol). | | | | | | | | |

| **Table S6** Results of multiple linear regression analyses of the associations between self-reported hopefulness and overall cognitive function according to physical activity status in older adults without major depression (N = 117) | | | | | | |  |
| --- | --- | --- | --- | --- | --- | --- | --- |
|  |  |  | Total score of CERAD | | | | |
|  |  |  | B | SE | $\beta$ | *p* | |
| *Low physical activity (n = 36)* | | | | | | | |
| Model 1 ^a^ |  |  |  | | |  | |
| Hopefulness |  |  | 5.113 | 4.265 | 0.201 | 0.239 | |
| Non-hopefulness |  |  | Reference | | |  | |
| Model 2 ^b^ |  |  |  | | |  | |
| Hopefulness |  |  | 2.837 | 4.765 | 0.112 | 0.559 | |
| Non-hopefulness |  |  | Reference | | |  | |
| *High-to-moderate physical activity (n = 81)* | | | | | | | |
| Model 1 ^a^ |  |  |  | | |  | |
| Hopefulness |  |  | 6.907 | 1.996 | 0.273 | <0.001 | |
| Non-hopefulness |  |  | Reference | | |  | |
| Model 2 ^b^ |  |  |  | | |  | |
| Hopefulness |  |  | 5.009 | 1.667 | 0.163 | 0.003 | |
| Non-hopefulness |  |  | Reference | | |  | |
| CERAD Consortium to Establish a Registry for Alzheimer’s Disease, APOE4 apolipoprotein ε4, VRS vascular risk score, BMI body mass index, HDL high density lipoprotein, LDL low density lipoprotein.  ^a^ Crude model (no adjustment for covariates).  ^b^ Model adjusted for age, sex, education, APOE4, VRS, BMI, annual income, alcohol intake, smoking, dietary habits (protein, fruit and vegetable intake), and blood-based nutritional markers (albumin, glucose, HDL, and LDL cholesterol). | | | | | | |  |
